# Transmission of *Babesia microti* Parasites by Solid Organ Transplantation

**DOI:** 10.3201/eid2211.151028

**Published:** 2016-11

**Authors:** Meghan B. Brennan, Barbara L. Herwaldt, James J. Kazmierczak, John W. Weiss, Christina L. Klein, Catherine P. Leith, Rong He, Matthew J. Oberley, Laura Tonnetti, Patricia P. Wilkins, Gregory M. Gauthier

**Affiliations:** University of Wisconsin School of Medicine and Public Health, Madison, Wisconsin, USA (M.B. Brennan, J.W. Weiss, C.L. Klein, C.P. Leith, R. He, M.J. Oberley, G.M. Gauthier);; Centers for Disease Control and Prevention, Atlanta, Georgia, USA (B.L. Herwaldt, P.P. Wilkins);; Wisconsin Division of Public Health, Madison (J.J. Kazmierczak);; American Red Cross Badger–Hawkeye Blood Service Region, Madison (J.W. Weiss);; American Red Cross Jerome H. Holland Laboratories for the Biomedical Sciences, Rockville, Maryland, USA (L. Tonnetti)

**Keywords:** babesiosis, human babesiosis, Babesia microti, parasites, protozoa, organ transplantation, kidney transplant, erythrocyte transfusion, tissue donor, hemolytic anemia, vector-borne infections, Wisconsin, Minnesota, United States

## Abstract

*Babesia microti*, an intraerythrocytic parasite, is tickborne in nature. In contrast to transmission by blood transfusion, which has been well documented, transmission associated with solid organ transplantation has not been reported. We describe parasitologically confirmed cases of babesiosis diagnosed ≈8 weeks posttransplantation in 2 recipients of renal allografts from an organ donor who was multiply transfused on the day he died from traumatic injuries. The organ donor and recipients had no identified risk factors for tickborne infection. Antibodies against *B. microti* parasites were not detected by serologic testing of archived pretransplant specimens. However, 1 of the organ donor’s blood donors was seropositive when tested postdonation and had risk factors for tick exposure. The organ donor probably served as a conduit of *Babesia* parasites from the seropositive blood donor to both kidney recipients. Babesiosis should be included in the differential diagnosis of unexplained fever and hemolytic anemia after blood transfusion or organ transplantation.

## Abstract 2

Infection with this parasite should be included in differential diagnosis of fever and anemia after blood transfusion or organ transplantation.

*Babesia microti*, an intraerythrocytic parasite, is the most common cause of human babesiosis in the United States and is endemic to the Northeast and upper Midwest regions, including parts of Wisconsin and Minnesota (*1**–**4*). *B. microti* infection can range from asymptomatic to severe. Common manifestations include hemolytic anemia and nonspecific influenza-like symptoms (*2*). Persons who are asplenic, elderly, or immunocompromised are at increased risk for symptomatic infection and for severe complications, such as multiorgan dysfunction and death (*5*).

The primary route of transmission of *B. microti* parasites is by the bite of an infected *Ixodes scapularis* tick (*6*). Transmission of *Babesia* parasites by blood transfusion also is well-documented (*7**–**11*). In contrast, transmission associated with solid organ transplantation has not been reported. We investigated 2 cases of babesiosis for which transmission probably occurred when renal allografts were transplanted from a multiply transfused organ donor.

## Materials and Methods

Diagnosis of babesiosis in 2 persons who received kidney transplants from the same donor prompted multifaceted, collaborative investigations, which were conducted by the authors and acknowledged persons and agencies (e.g., transplant, transfusion, and public health organizations). The Organ Procurement Organization (Madison, WI, USA) identified the disposition of all organs and tissues recovered from the organ donor and notified the United Network for Organ Sharing (Richmond, VA, USA) about the possibility of donor-derived transmission.

Only kidneys and corneas had been transplanted; the bilateral iliac arteries and veins had been recovered but discarded 14 days later, whereas the liver and other tissues that had been donated for research were embargoed. Medical and transfusion records of the organ donor and transplant recipients were reviewed, as were procedures and records for organ/tissue recovery, handling, and transplantation. The transplant recipients, the seropositive blood donor identified in the transfusion investigation, and surrogates for the organ donor were interviewed regarding risk factors for and potential clinical manifestations of *Babesia* infection.

Specimens from the transplant recipients, the organ donor, and the organ donor’s blood donors were tested for evidence of *Babesia* infection. Evaluations of the transplant recipients included light microscopy of Giemsa- or Wright-stained thick and thin blood smears for *Babesia* parasites. The Centers for Disease Control and Prevention (CDC; Atlanta, GA, USA) conducted reference diagnostic testing of specimens from transplant recipients and organ donor. CDC also conducted serologic testing by using an indirect fluorescent antibody (IFA) assay for total immunoglobulin against *B. microti* antigens (*12*). Serum and plasma specimens were tested in serial 4-fold dilutions, and a reciprocal dilution titer of 64 was considered positive. CDC conducted PCR analysis of whole-blood specimens from the transplant recipients by using primers specific for the *B. microti* 18S rRNA gene (*13*) and a previously described 2-step nested PCR (*7*). CDC also conducted *B. microti* PCR analysis of fresh-frozen hepatic tissue from the organ donor. No fresh-frozen renal tissue or whole-blood specimens from the organ donor were available for testing. However, paraffin-embedded, pretransplantation specimens from both kidneys were available and were tested by using a *B. microti* immunohistochemical (IHC) assay (*14*); CDC also conducted IHC testing of hepatic tissue.

The American Red Cross obtained blood/serum specimens from all 33 blood donors who had contributed components transfused into the organ donor. No segments or components from original donor units were available for testing. The American Red Cross tested postdonation specimens by using a *B. microti* IFA assay for IgG and a *B. microti* real-time PCR. IFA testing was conducted with serial 2-fold dilutions of samples.

## Case Reports

### Renal Transplant Recipients

In late August 2008, two men with end-stage diabetic nephropathy (a 65-year-old Wisconsin resident [patient A; the index case-patient] and a 41-year-old Iowa resident [patient B]) received renal allografts from the same deceased donor at the University of Wisconsin Hospital and Clinics (UWHC; Madison, WI, USA). Different surgeons in separate operating rooms transplanted the kidneys. Both patients received induction immunosuppressive therapy with basiliximab and maintenance therapy with prednisone, mycophenolate mofetil, and tacrolimus. During the previous year and peritransplant period, neither patient lived or traveled in babesiosis-endemic regions, which in the Midwest, included parts of Minnesota and Wisconsin but not Iowa (Table), and they did not receive blood transfusions.

**Table:** Characteristics of 2 patients who received renal allografts from the same organ donor and became infected with *Babesia microti* parasites, 2008*

Characteristic	Patient A (index case-patient)	Patient B
Type of kidney transplant	Left	Right
Age, y/sex	65/M	41/M
Residence†	Southcentral Wisconsin (urban, nonwooded area of Sauk County)	Iowa (semirural area bordering southwestern Wisconsin)
Cause of end-stage nephropathy	Type 2 diabetes mellitus	Type 1 diabetes mellitus
Pretransplant dialysis	Peritoneal dialysis in Wisconsin	Hemodialysis in Iowa
Other medical history	Diabetic retinopathy; coronary artery disease	Diabetic retinopathy (legally blind); hypertension
Duration of hospitalization for renal transplantation, d‡	6 (late Aug–early Sep)	10 (late Aug–early Sep; patient had moderate delay in graft function)
Clinical manifestations potentially attributable to babesiosis	Fever (39.4°C), sweats, fatigue, anorexia, dark urine	Fever (38°C), fatigue, abdominal pain
*Babesia* blood-smear examination		
Date of first positive blood smear	Oct 20	Oct 23
Initial parasitemia level, %	8	1
Context for diagnosis	Platelet clumping prompted manual (nonautomated) review of blood smear	Diagnosis of case in patient A prompted evaluation of patient B during a routine clinic visit
Date of last positive blood smear	Oct 24	Oct 23
Date of last *B. microti* PCR-positive blood specimen§	Nov 7	Nov 21
*B. microti* IFA titer (date)		
Pretransplant serum sample	<8 (Jul 30)	<8 (Aug 11)
Posttransplant serum sample	4,096 (Oct 21)	1,024 (Oct 23)
Laboratory values when babesiosis was diagnosed (2, 6, and 16 wks after initiation of therapy)¶		
Hematocrit, %#	21 (21, 45, 49)	35 (41, 37, 44)
Reticulocyte, %	11.7	4.3
Leukocyte count, x 10^9^/L**	6.7	5.5
Platelet count, x 10^9^/L	157	154
Haptoglobin, mg/dL	<8 (24, 67, 104)	ND (154, 223, 202)
Lactate dehydrogenase, U/L	747 (490, 220, ND)	495 (365, 344, 331)
Creatinine, mg/dL	1.1	1.3
Dates of hospitalization for babesiosis	Oct 20–24	None
Dates of 6-wk course of azithromycin and atovaquone	Oct 20–Dec 1	Oct 23–Dec 4

*IFA, indirect fluorescent antibody; ND, not done. †Neither patient had lived or traveled in babesiosis-endemic areas in Wisconsin (primarily, the northwestern and northcentral regions) or elsewhere. ‡Preparation of kidneys for transplantation included an in situ flush (initiated 25 min after the donor was declared brain dead and was extubated) with 2 L of University of Wisconsin solution (*15*), each of which was infused in <4 min; a flush with 200 mL of this solution after the kidneys were explanted; and continuous circulation with kidney perfusate solution until the kidneys were transplanted. §Both patients had negative PCR results for followup blood specimens in February 2009. ¶Reference ranges: creatinine, 0.6–1.3 mg/dL; haptoglobin, 30–200 mg/dL; lactate dehydrogenase, 90–200 U/L. #Hematocrit values posttransplantation were 37% (patient A) and 40% (patient B). **Differential leukocyte counts were 73% neutrophils, 14% lymphocytes, and 13% monocytes for patient A; and 79% neutrophils, 12% lymphocytes, 8% monocytes, 1% eosinophils, and 1% basophils for patient B.

**Figure 1:**
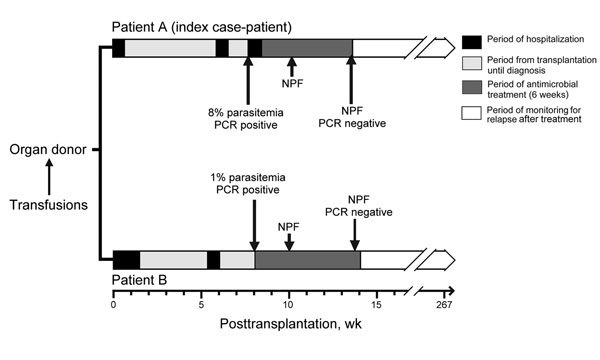
Timelines showing key clinical and laboratory events for 2 renal transplant recipients (patients A and B) infected with *Babesia microti* parasites, Wisconsin, USA, 2008. Trauma, transfusions, death, and organ procurement for the organ donor all occurred on the same day in late August 2008. NPF, no parasites were found by examination of thick and thin blood smears.

Both patients showed seroconversion and development of parasitologically confirmed cases of babesiosis, which were diagnosed ≈8 weeks posttransplantation (Table; Figure 1). At the request of the transplant physicians, both patients were evaluated by the same UWHC infectious disease specialists.

After babesiosis was diagnosed, doses of immunosuppressive medications were decreased and each patient received a 6-week course of oral antimicrobial drug therapy: atovaquone (750 mg, 2×/d for 6 wks) plus azithromycin (1,000 mg, 1×/d for 2 wks, followed by 600 mg, 1×/d for 4 wks). During therapy, symptoms resolved, laboratory parameters returned to reference ranges or values, and *Babesia* parasite DNA became undetectable (Table; Figure 1).

#### Patient A

On October 2, 2008 (≈5 weeks posttransplantation), during a routine follow-up appointment at the UWHC Transplant Clinic, the wife of patient A mentioned that he had a lack of energy and decreased appetite (onset date not specified). At that clinic visit, his hematocrit was 37%, which approximated his baseline value posttransplantation.

On October 8, he was admitted to the UWHC, as planned, to have his peritoneal dialysis catheter removed the next day. However, at admission, he unexpectedly was found to have a temperature of 39.4°C. His hematocrit values were 33% and 28% on October 8 and 9, respectively. Removal of the catheter was postponed until October 10, and he was discharged after the procedure. Cultures of the catheter tip, blood, and urine specimens were negative for bacterial growth. He was treated empirically with piperacillin/tazobactam during his 2-day hospitalization, followed by a 7-day outpatient course of ciprofloxacin.

**Figure 2:**
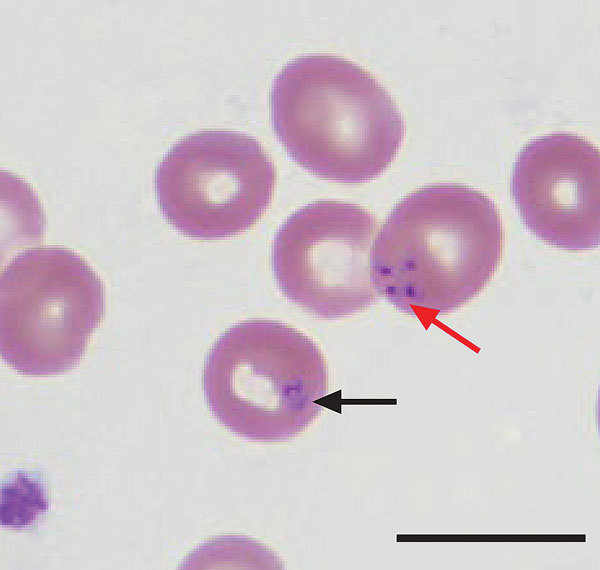
Wright-stained peripheral blood smear from patient A (index case-patient), a renal transplant recipient infected with *Babesia microti* parasites, Wisconsin, USA, 2008. The smear shows intraerythrocytic *Babesia* parasites, a ring form (black arrow), and a Maltese cross or tetrad form (red arrow), which is pathognomonic for babesiosis. Scale bar indicates 10 μm.

On October 16 (day 6 of ciprofloxacin therapy), his wife called the transplant coordinator to report that he had a low-grade temperature (37.5°C) and a 2-day history of drenching sweats. An appointment in the Transplant Clinic was scheduled for October 20 to evaluate his symptoms. During the appointment, he reported a several-day history of darkening urine and progressive fatigue since his previous hospitalization. Per routine for clinic visits, a complete blood count was determined. His hematocrit had decreased to 21% (Table). Because platelet clumping was detected by using an automated hematology analyzer, a blood smear was reviewed manually: intraerythrocytic *Babesia* parasites were visualized at a parasitemia level of 8% (Table; Figure 2). On the same day (October 20), he was admitted to the UWHC, evaluated by the Infectious Disease Service, and began treatment with azithromycin plus atovaquone (Table; Figure 1). Within 48 hours of initiating therapy, his appetite and exercise tolerance increased, and his parasitemia level decreased to <5%.

#### Patient B

During October 4–9, patient B was hospitalized in Iowa for evaluation of epigastric discomfort, dyspepsia, nausea, and low-grade fever of unclear etiology. His hemoglobin level was 12.1 g/dL. Computed tomography (CT) imaging of his abdomen and pelvis was unremarkable except for enlargement of the pancreatic head (amylase and lipase values were within reference ranges). While hospitalized, he was treated empirically with metronidazole and levofloxacin; a 7-day outpatient course of ciprofloxacin therapy was prescribed.

**Figure 3:**
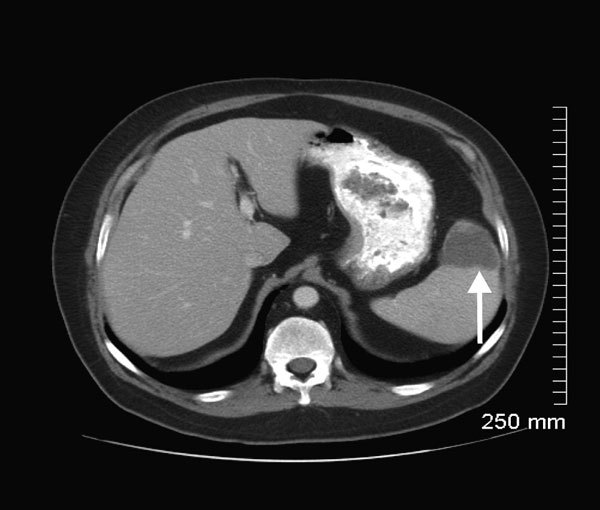
Computed tomography (CT) scan of the abdomen of patient B, a renal transplant recipient infected with *Babesia microti* parasites, Wisconsin, USA, 2008. Taken on November 5, the scan shows a splenic infarction (white arrow) that had not been visualized on a CT scan on October 5. Although the cause of the splenic infarction was not determined, the infarction might have been a complication of babesiosis, as reported for other patients (*16**,**17*).

On October 23, during a routine follow-up appointment in the UWHC Transplant Clinic, he was afebrile but, on prompting, recalled a transient fever (38°C) ≈1 week earlier. In addition, he reported a several-week history of left upper quadrant pain. At examination, he had tenderness to deep palpation of the left upper quadrant, which worsened with deep inspiration. A manual (nonautomated) review of a blood smear was requested explicitly, prompted by diagnosis of the case of babesiosis in patient A 3 days earlier. Intraerythrocytic *Babesia* parasites also were observed on the blood smear for patient B; the parasitemia level was 1%. His hemoglobin level was 11 g/dL, and his hematocrit was 35%. On the same day, he was evaluated in the UWHC Infectious Disease Clinic and began outpatient therapy with atovaquone plus azithromycin. To evaluate his abdominal pain, CT of the abdomen and pelvis was performed on an outpatient basis (November 5); it showed a splenic infarction (Figure 3), which was not detected by CT in early October. During the course of antimicrobial drug therapy, his abdominal pain and constitutional symptoms resolved.

### Organ Donor

The organ donor was a 22-year-old man who was a resident of an urban area of Wisconsin to which babesiosis was not endemic. According to his relatives and primary care physician, he had been in good health and did not have any potentially relevant travel or clinical manifestations during the previous year. His only known risk factor for exposure to *Babesia* parasites was receipt of multiple blood transfusions during resuscitation attempts on the day he died from unintentional trauma. Although an autopsy was not performed, a limited number of plasma, serum, and tissue specimens were available for *Babesia* testing. Antibodies against *B. microti* parasites were not detected by retrospective serologic testing of a pretransfusion plasma specimen and 2 posttransfusion serum specimens (IFA titer <8). Tissue sections from both kidneys had negative IHC results. IHC testing of hepatic tissue showed a few rare foci of suspicious staining but no definitive evidence of *Babesia* parasites, and hepatic tissue showed negative results by PCR.

The cornea recipients were contacted, and blood specimens collected ≈3–4 months posttransplantation were tested for evidence of *Babesia* infection. Specimens showed negative results for PCR and IFA analysis, and no parasites were found on blood smears.

### Transfusion Investigation

During resuscitation attempts, the organ donor received 20 cellular blood components (19 units of erythrocytes and 1 unit of apheresis platelets) and 13 plasma units. Only 1 of the 33 donors, a 52-year-old man, had evidence of *B. microti* infection. Specimens available for testing were collected 88 and 151 days postdonation and had IFA titers of 256 and 128, respectively; both specimens showed negative results by PCR. The seropositive blood donor was the source of 1 of the organ donor’s last erythrocyte transfusions, which was transfused 15 days postdonation.

This blood donor lived in a babesiosis-endemic area of Minnesota (Washington County) and had camped in disease-endemic areas in northern Wisconsin (Ashland County) in May 2008 and in northern Minnesota (Saint Louis County) in July 2008. During the retrospective investigation, he recalled a fever (39.4°C), chills, and diaphoresis, which lasted ≈36 hours, during the first week of June. Although he did not recall any tick bites, his wife reportedly had found a tick on his body (timing and other details not specified). No cellular components from his donation in August were transfused to other patients. After he was found to be seropositive, he was deferred indefinitely from future blood donations. However, he already had donated blood in the interim (in September 2008), and apheresis platelets from the donation had been transfused. A specimen obtained ≈2 months posttransfusion from the platelet recipient was tested in a commercial laboratory and showed negative *B. microti* IFA and PCR results.

## Discussion

We investigated parasitologically confirmed cases of babesiosis in 2 recipients of renal allografts from an organ donor whose only known risk factor for exposure to *Babesia* parasites was the receipt of multiple blood transfusions on the day he died. The organ donor and the kidney recipients did not have antibodies against *B. microti* parasites detected by retrospective testing of pretransplantation specimens. However, 1 of the organ donor’s blood donors was seropositive when tested postdonation and had risk factors for tick exposure.

The most likely scenario is that the kidney donor served as a conduit of *Babesia* parasites from this blood donor to the kidney recipients (i.e., the blood donor became infected by tickborne transmission, secondary transmission occurred by erythrocyte transfusion, and tertiary transmission occurred by organ transplantation). The possibility that the kidney recipients became infected independently is remote: they did not live, travel, or receive medical care in any known babesiosis-endemic areas in the Midwest or elsewhere; they did not receive any transfusions; and they showed seroconversion posttransplantation, despite being immunosuppressed. Although no subtyping tools are available to establish that the patients were infected with the same *B. microti* strain, they almost assuredly became infected from the same source at approximately the same time.

Previous reports have described organ transplant recipients who became infected with *Babesia* parasites by tickborne- or transfusion-associated transmission in the peritransplant period or thereafter (*10**,**18**–**22*). Transplantation-associated transmission of *B. microti* parasites, which are not known to have an exoerythrocytic tissue phase, has not been described, nor has the occurrence of 3 consecutive routes of transmission (vector, transfusion, and transplantation), which has been reported for West Nile virus (*23**,**24*). The plausibility of transplantation-associated transmission of *B. microti* parasites, in the context of residual parasites in the renal vasculature/fluids after flushing the organs, is supported in part by data from other contexts (e.g., transfusion-associated cases) that suggest low inocula of the parasite can cause infection (*10**,**25*).

Although we do not have proof that the blood donor was infected when he donated blood or have laboratory evidence that the organ donor briefly harbored the parasite, the negative PCR results for the postdonation specimens from the blood donor and the negative PCR and IHC results for the available posttransfusion specimens from the organ donor are not helpful; only positive results would have been informative. Although other transmission scenarios seem much less probable, the cases of babesiosis in the kidney recipients we report would be noteworthy even if the organ donor recently had acquired the parasite from a tick (i.e., was in the early window period of infection, despite his lack of known risk factors for tickborne transmission).

Diagnosis of babesiosis in the kidney recipients prompted multiagency investigations of the organ donor and his blood donors. However, the cases of babesiosis in the recipients could have been easily missed, which highlights the possibility that other transplantation-associated cases have occurred but were not diagnosed or investigated. For patient A (index case-patient), babesiosis was diagnosed because of the serendipitous finding of parasites on a blood smear that was examined manually because of platelet clumping. His lack of risk factors for tickborne transmission and the possibility of donor-derived infection led to prompt evaluation of patient B. At the time of diagnosis, illness in patient B was milder (lower-level parasitemia, minimal anemia, and transient fever) than that in patient A, even though the 2 patients received similar immunosuppressive regimens.

Babesiosis can be persistent, relapsing, or life threatening in immunocompromised patients (*18**,**19**,**22**,**26**–**28*). Optimal therapy for babesiosis in patients who have received an organ transplant or have impaired immunity for other reasons is not well established and might depend on multiple factors; a uniform recommendation might not be applicable to such a heterogeneous population. In immunocompetent persons, the typical duration of antimicrobial drug therapy for babesiosis is 7–10 days (*6*). We decided to treat both kidney recipients for 6 weeks on the basis of retrospective data for immunosuppressed patients that suggest the likelihood of cure is higher if combination antimicrobial drug therapy is administered for >6 weeks, including 2 weeks after *Babesia* parasites are no longer detected on blood smears (*27*). We gave the patients atovaquone plus azithromycin rather than clindamycin plus quinine (the standard of care for severely ill patients [*6**,**29*]) to minimize the likelihood of toxicity during their 6-week treatment courses. In addition, we decreased the doses of their immunosuppressive medications.

Both patients tolerated and responded well to the antimicrobial treatment, without documented relapses. However, clinicians should be aware that clinical resistance reportedly developed in several immunosuppressed patients treated for prolonged periods with atovaquone plus azithromycin (*28*); whether particulars of those patients’ treatment regimens (e.g., antimicrobial drug dosing) contributed to development of clinical resistance is not known (*28*,*30*).

The cases of babesiosis we describe not only underscore the plausibility and likelihood of transmission by organ transplantation, but also highlight the emerging role of transfusion-associated babesiosis. For the 3-decade period of 1979 (the year the first known transfusion case occurred) through 2009, a total of 159 transfusion-associated cases of *B. microti* infection were identified in the United States, most (77%) of which occurred during 2000–2009 (*10*). Asymptomatic persons can fulfill all of the criteria for donating blood despite having low-level parasitemia sufficient to cause infection in a transfusion recipient (*10*).

To date, no *Babesia* tests for screening US blood donors have been licensed by the Food and Drug Administration, and no pathogen-reduction technologies for cellular blood components have been approved (*31**–**35*). However, the Blood Products Advisory Committee of the Food and Drug Administration that was convened on May 13, 2015, supported the concepts of year-round *B. microti* serologic testing of all US blood donors and of *B. microti* nucleic acid–based testing of donors in selected states (details remain to be determined) (*36*). Because of donor travels and shipments/distributions of blood components, transmission by transfusion is not limited to babesiosis-endemic foci (*10*). For example, the seropositive erythrocyte donor we identified had donated blood in a babesiosis-endemic area of Minnesota. This blood was then transported to and transfused in an area of Wisconsin to which babesiosis was not endemic.

As we described, unrecognized tickborne transmission of *Babesia* parasites to the blood donor probably led to transmission by transfusion to the organ donor and subsequent transmission by organ transplantation to both kidney recipients. Clinicians should include babesiosis in the differential diagnosis of unexplained fever and hemolytic anemia after blood transfusion or organ transplantation, even in regions to which babesiosis is not endemic. Suspected cases of iatrogenic transmission should be reported to state and local public health authorities. In addition, cases that might be transfusion or transplantation associated should be reported to the pertinent blood center and organ procurement organization, respectively.
